# Calcitonin gene-related peptide (receptor) antibodies: an exciting avenue for migraine treatment

**DOI:** 10.1186/s13073-018-0524-7

**Published:** 2018-02-22

**Authors:** Antoinette MaassenVanDenBrink, Gisela M. Terwindt, Arn M. J. M. van den Maagdenberg

**Affiliations:** 1 Department of Internal Medicine, Division of Vascular Medicine and Pharmacology, Erasmus MC, Rotterdam, The Netherlands; 2 Department of Neurology, Leiden University Medical Centre, Leiden, The Netherlands; 3 Department of Human Genetics, Leiden University Medical Centre, Leiden, The Netherlands

## Abstract

Specific prophylactic migraine treatments are urgently needed because of the unmet needs of many migraine patients. Antibodies targeting calcitonin gene-related peptide (CGRP) or its receptor have recently shown efficacy in episodic and chronic migraine and will be available soon.

## Why do we need new drugs for migraine?

Migraine is a debilitating episodic brain disorder affecting about 15% of the population. Migraine attacks typically consist of severe, unilateral headaches that are accompanied by nausea, vomiting and photo- and phonophobia, lasting 4–72 h [1]. The median attack frequency is 1.5 per month, but many patients suffer from weekly attacks. Migraine is a multifactorial genetic disorder for which several dozen gene variants, all with small effect size, have been identified that suggest the involvement of neuronal and vascular mechanisms in disease pathology [2]. Similar disease mechanisms, albeit involving different genes, have surfaced in rare monogenic familial hemiplegic migraine (FHM) and in various monogenic syndromes in which migraine is very prevalent among mutation carriers, for example, familial advanced sleep-phase syndrome (FASPS) and cerebral autosomal dominant arteriopathy with subcortical infarcts and leukoencephalopathy (CADASIL) [2].

Specific acute migraine treatment improved three decades ago with the advent of the ‘triptans’—5-hydroxytryptamine_1_ (5-HT_1_) receptor agonists—but not all patients respond adequately. Attack frequency may increase with overuse of acute headache medication, resulting in a transition from episodic to chronic migraine (defined as 15 or more headache days per month with at least 8 migraine days). Activation of the trigeminovascular system seems pivotal in the generation of attacks. Basic and clinical research revealed that specific molecules, such as calcitonin gene-related peptide (CGRP), are increased during attacks, which make them potential targets for preventive drug development [1, 3].

## Calcitonin gene-related peptide and its receptor: drug targets for the treatment of migraine

CGRP is a 37-amino-acid neuropeptide that, together with its receptor, is located in both the central and the peripheral nervous system. Besides being a neuromodulator, CGRP is one of the most potent vasodilators known. The canonical CGRP receptor consists of three components: calcitonin-like receptor (CLR; a seven-transmembrane receptor component), receptor activity modifying protein 1 (RAMP1), and receptor component protein (RCP) [3]. The involvement of CGRP in migraine was suggested and demonstrated about 30 years ago by Edvinsson and Goadsby (see [3]). Since then, several attempts have been made to develop antimigraine drugs that inhibit the actions of CGRP. The first approach was the development of small molecule CGRP receptor antagonists, the so-called ‘gepants’. These molecules, which are competitive receptor antagonists, were all effective in the acute treatment of migraine, and some were successfully tested for the prophylactic treatment of migraine. Unfortunately, due to pharmacokinetic and toxicity issues, none of the gepants has reached the clinic [4]. However, several new gepants are in the clinical phase of development (reviewed in [3, 4]).

Apart from the gepants, antibodies against CGRP (eptinezumab, fremanezumab, and galcanezumab, which are humanized antibodies) or the CGRP receptor (erenumab, a fully human antibody) have been developed recently. Because of their pharmacokinetic properties—parenteral administration with a long time to achieve maximal drug concentration (T_max_) and a long plasma elimination half-life (T_1/2_)—these drugs are intended for the prophylactic treatment of migraine. Clinical trials on all four antibodies have been positive and the tolerability of the antibodies is excellent, with an adverse event profile similar to that of placebo (see [4]). Recently, the results of two different phase 3 trials on two of these antibodies were reported [5, 6].

Goadsby and colleagues [5] described a trial on the CGRP receptor antibody erenumab in a population of 955 migraine patients with episodic migraine. Patients received subcutaneous injections of either 70 or 140 mg erenumab, or placebo, monthly. The primary end point was a change in mean migraine days per month from baseline to months 4 through 6. At baseline, the overall average of migraine days was 8.3 per month. Both doses of erenumab significantly differed from placebo in the primary end point; the mean decrease in migraine days per month was 3.2 (70 mg) and 3.7 (140 mg) days in the erenumab group and 1.8 days in the placebo group. A ≥ 50% reduction in the mean number of migraine days per month was achieved for 43% (70 mg) and 50% (140 mg) of patients when compared to placebo (27%).

Silberstein and colleagues [6] performed a trial with the CGRP antibody fremanezumab in 1130 patients with chronic migraine. Patients received subcutaneous injections of fremanezumab, in either a quarterly (675 mg at baseline and placebo at weeks 4 and 8) or a monthly (675 mg at baseline and 225 mg at weeks 4 and 8) dosing regimen, or matching placebo. The primary end point was the mean change from baseline in the average number of headache days per month during the 12 weeks after the first dose. The mean number of baseline headache days was 13 per month. The mean reduction in headache days per month was 4.3 and 4.6 for fremanezumab administered quarterly or monthly, respectively, and 2.5 for placebo. A ≥ 50% reduction in the mean number of headache days per month was achieved for 38% (quarterly) and 41% (monthly) of patients when compared to placebo (18%).

In accordance with earlier trials, the side effects were similar for erenumab or fremanezumab and placebo. Although these two trials differ in their patient populations (episodic vs. chronic migraine), design, and primary end point, the results seem to indicate a consistent decrease in headache burden after the use of the antibodies. Nevertheless, the therapeutic gain vs. placebo (16–23%) is small.

## CGRP mechanisms and challenges

It is interesting to speculate whether there is a clinically relevant difference between blockade of the receptor (erenumab) or blockade of CGRP itself (eptinezumab, fremanezumab, galcanezumab). As we described before [7], this could theoretically be the case because peptides other than CGRP could bind to the CGRP receptor when CGRP-binding antibodies are used, and CGRP might act at receptors other than the CGRP receptor when the CGRP receptor-binding antibody is used. Indeed, the amylin_1_ receptor (calcitonin receptor (CTR) instead of CLR coupled to RAMP1 and RCP) was recently described to act as a functional CGRP receptor in the trigeminal system, and probably also in the vasculature [7]. However, there is no evidence at present to confirm or refute whether there will be a clinically meaningful difference between these two different modes of action.

Another relevant question is where the site of action of the antibodies is located. Because of the large molecular size of the antibodies (molecular weight of ~ 150 kDa), they are unlikely to cross the blood–brain barrier (BBB) in significant amounts. Thus, their point of action will most probably be located outside the BBB and could include a vascular site, or neuronal structures that are not protected by the BBB, such as the trigeminal ganglion and the paraventricular structures. Indeed, a vascular action for CGRP seems to be present in, for example, the protective mechanism against ischemia (which is relevant in view of cardiovascular safety [7]) or hypertension, as has been demonstrated in CGRP-knockout mice that showed enhanced hypertension in response to angiotensin II infusion [8]. CGRP might also affect the migraine phenotype via neuronal pathways, as evident from experiments in mice overexpressing RAMP1 neuronally [9]. A clear distinction between the neuronal and vascular components is difficult, as there seems to be an intensive crosstalk between these two systems [10].

## Conclusions

The advent of CGRP (receptor)-binding antibodies represents a valuable novel treatment option for migraine. In contrast to current prophylactic antimigraine drugs, this is the first class specifically developed for the treatment of migraine. Although long-term safety remains to be confirmed, we consider the arrival of the antibodies as a very positive development. The emergence of this novel class of drugs is good news, but it is fair to state that blockade of the CGRP pathway does not seem to be a panacea for all migraine patients, as response rates are not perfect. Future research should focus on identifying characteristics of patients who do not respond to CGRP (receptor) blockade, for example, genetic factors that determine response. In non-responders, other pharmacological targets might be explored to establish proper reduction of migraine attacks. Besides CGRP, other modulators of the trigeminovascular system may be of relevance in migraine. For example, pituitary adenylate cyclase-activating peptide (PACAP) and vasoactive intestinal peptide (VIP) have been described as being involved in headache pathophysiology. Novel drugs that are focused on these targets are currently being developed [11].

## Abbreviations

BBB: Blood–brain barrier
CGRP: Calcitonin gene-related peptide
CLR: Calcitonin-like receptor
RAMP1: Receptor activity modifying protein 1
RCP: Receptor component protein
